# ZC3H12A: A Critical Mediator of Inflammation, Tumor Immunotherapy, and Metabolic–Immune Crosstalk—Implications for Disease Treatment

**DOI:** 10.3390/biom15101473

**Published:** 2025-10-19

**Authors:** Mingjun Lu, Jingwei Guo, Chenyang Wang, Bingbing Wan, Teng Ma

**Affiliations:** 1 Cancer Research Center, Beijing Chest Hospital, Capital Medical University, Beijing Tuberculosis and Thoracic Tumor Research Institute, Beijing 101149, China; L564347411@163.com (M.L.); gjw007@mail.ccmu.edu.cn (J.G.); tb2023042003@pumc.edu.cn (C.W.); 2 Key Laboratory of Systems Biomedicine (Ministry of Education), Shanghai Center for Systems Biomedicine, Shanghai Jiao Tong University, Shanghai 200240, China

**Keywords:** ZC3H12A, ribonuclease, inflammation, tumor immunotherapy, CAR-T

## Abstract

ZC3H12A is a key RNA-binding protein and ribonuclease that plays a central role in negatively regulating inflammation and maintaining immune homeostasis. It does this by degrading the mRNA of multiple inflammatory mediators (such as *IL-6* and *IL-1β*), as well as through its deubiquitinating enzyme activity. Not only does it limit excessive immune activation by regulating innate and adaptive immune cells (e.g., macrophages and T cells), but it also exerts bidirectional effects in tumors, acting as an anti-tumor factor to inhibit angiogenesis and oncogenic signal pathways, while promoting tumor progression under specific conditions. In recent years, ZC3H12A has emerged as a critical target for tumor immunotherapy, particularly CAR-T cell therapy. Its knockout significantly enhances T-cell persistence and anti-tumor efficacy, demonstrating broad translational potential. Furthermore, ZC3H12A plays a crucial role in systemic metabolic–immune crosstalk and infectious diseases. This review systematically summarizes the multifunctional roles of ZC3H12A in immune regulation, tumor therapy, metabolic disorders and inflammation-related diseases, with the aim of providing new insights into its potential application in the treatment of human diseases.

## 1. Introduction

Chronic inflammation is a critical link between intrinsic factors, such as oncogenes and tumor suppressor genes, and extrinsic factors, such as immune and stromal components, in tumor development [1]. It promotes repeated tissue damage and repair, increases mitotic errors, and facilitates the accumulation of cancer-prone cells, with epidemiological studies implicating it in 15–20% of cancers [2,3]. Inflammatory responses are governed by the dynamic regulation of gene expression in immune cells, in which RNA metabolism, especially post-transcriptional control, plays a pivotal role. Toll-like receptors (TLRs) recognize pathogens and trigger signaling that induces immune gene transcription and cytokine production [4,5]. Additionally, post-transcriptional mechanisms, mediated by RNA-binding proteins and specific nucleases, regulate mRNA stability and translation by targeting cis-elements, such as AU-rich elements and stem-loop structures in the 3′ UTR, thereby preventing excessive inflammation [6,7,8,9].

ZC3H12A, as an RNA-binding protein (RBP), plays a crucial role in regulating immune homeostasis and inflammatory factors [10]. In 2006, MCP-1-induced protein (MCPIP) was first identified in MCP-1-stimulated human monocytes as a novel protein derived from a highly induced, previously uncharacterized expressed sequence tag (EST), localized to the cell nucleus, and induced cardiomyocyte apoptosis [11,12]. Subsequent studies mapped this EST to the cDNA clone AW206332 (GenBank), identifying the gene *ZC3H12A* located on chromosome 1p34.3. It was proposed that *ZC3H12A* could bind cloned fragments of the cdh12 and cdh19 genes with DNA sequence specificity, implying a potential role as a transcriptional regulator In 2008, Liang et al. reported that lipopolysaccharide (LPS) induced the expression of ZC3H12A in macrophages [13]. They further identified a gene family comprising *ZC3H12A*, *ZC3H12B*, *ZC3H12C*, and *ZC3H12D*, all of which contain the CCCH zinc finger domain [14]. By 2009, ZC3H12A (also termed MCPIP1 or Regnase-1) was established as a ribonuclease that binds to the 3′ UTR of target mRNAs via its CCCH zinc finger domain and cleaves transcripts encoding inflammatory mediators, thereby playing an essential role in preventing autoimmunity diseases and regulating inflammation [15].

**Figure 1:**
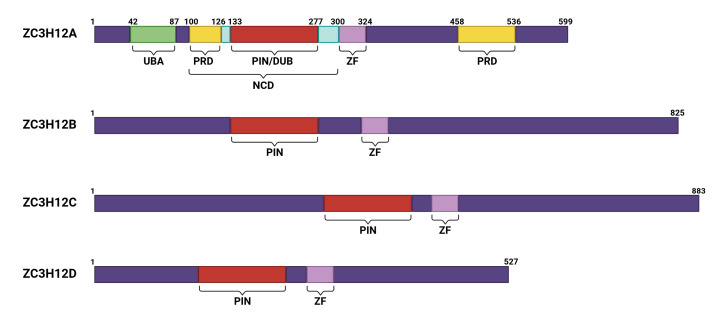
A schematic diagram of the CCCH zinc finger family members of ZC3H12A. All members of this family contain a CCCH zinc-finger domain. The domains in ZC3H12A were labeled as follows: UBA: 42-87; N-terminus PRD: 100-126; PIN/DUB: 133-277; NCD: 100-300; ZF: 300-324; N-terminus PRD: 458-536.

**Figure 2:**
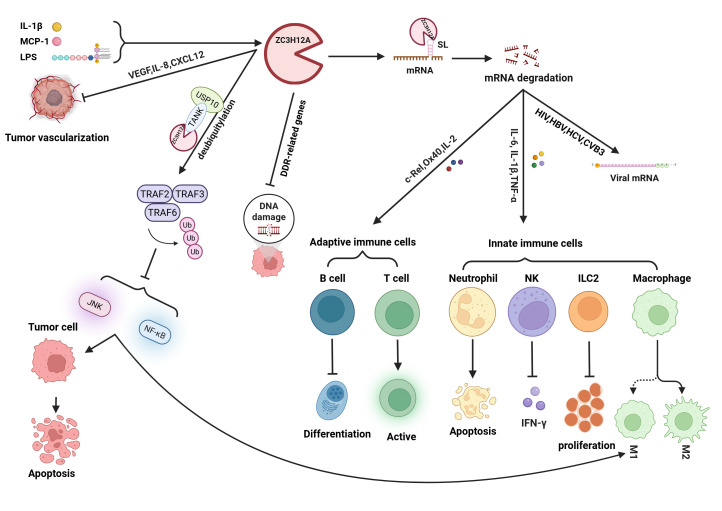
Ribonuclease ZC3H12A cleaves mRNA and regulates immune cells, tumor cells, and tumor angiogenesis, DNA damage repair, and molecular signaling pathways (NF-κB, JNK). Mode of action map. *ZC3H12A* regulates the development and function of innate and adaptive immune cells through RNase cleavage of various transcription factors (such as *Ox40* and *CXCL2*) and cytokines (such as *IL-6* and *IL-1β*), acting as a bridge between the innate and adaptive immune responses. Furthermore, RNase can cleave viral RNA, potentially offering a novel strategy for viral therapies. *ZC3H12A*’s DUB structure removes ubiquitin molecules from proteins, including TRAF2, TRAF3, and TRAF6, negatively regulating JNK and NF-κB activity, thereby controlling inflammation and promoting tumor apoptosis. ZC3H12A also influences DNA damage repair by regulating the expression of DNA damage-repair genes. In summary, ZC3H12A demonstrated significant potential in various human cancers and inflammatory diseases, making it a promising therapeutic target for future development.

The ZC3H12A protein consists of 599 amino acids and contains multiple domains with distinct functions (Figure 1) [16]. Among these, proline-rich domains (PRDs) are located at positions 100–126 (37% proline) and 458–536 (28% proline). These non-conservative domains significantly contribute to structural stability and functional activity by regulating conformational dynamics and protein interactions [12]. ZC3H12A also contains a CCCH-type zinc finger domain (ZF). CCCH zinc finger proteins constitute only 0.8% of all zinc finger proteins and possess RNA-binding capability [17,18]. Nearly 60 such proteins have been identified in humans and mice that modulate mRNA decay and immune signaling [19]. Unlike tristetraprolin (TTP) family proteins that target AU-rich elements [20,21], the ZF in ZC3H12A recognizes stem-loop (SL) structures and facilitates RNA degradation via hydrophobic stacking [9,22]. The PIN domain constitutes the RNase active site of ZC3H12A [14,23]. Key residues (Asp141, Asp225, Asp226, and Asp244) mediate Mg^2+^-dependent RNase activity [15,23], with Asp141 being essential [24]. Intramolecular interactions enable targeted degradation of inflammatory mRNAs (e.g., IL-6 and IL-1β) (Figure 2) [25]. Additionally, ZC3H12A contains a deubiquitinating enzyme domain (DUB) that overlaps with the PIN domain. Among the five major existing DUB families, it exhibits only distant homology with enzymes from the UCH family, indicating that it belongs to a novel category of DUBs [26,27,28]. This DUB activity enables ZC3H12A to deubiquitinate TRAF2, TRAF3, and TRAF6, thereby inhibiting JNK and NF-κB signaling and reducing the production of proinflammatory cytokines [26]. Furthermore, ZC3H12A forms a complex with USP10 via the adaptor protein TANK, enhancing its ability to remove polyubiquitin chains from NEMO and TRAF6, and suppress genotoxic *NF-κB* activation [29]. The physiological importance of this regulatory function is underscored by the hyperactivation of NF-κB observed in *ZC3H12A*-deficient mice [29,30].

Early studies primarily focused on describing the multifunctionality of ZC3H12A, including PRD-mediated protein interactions, ZF-dependent mRNA binding, PIN-driven RNA degradation, and DUB-mediated NF-κB regulation. These properties highlight its central role in post-transcriptional and post-translational regulation of inflammation and immune homeostasis [31]. However, recent research has been delving into the central role of ZC3H12A within complex disease networks—such as cancer, autoimmune diseases, and viral infections—while exploring its translational potential as a therapeutic target. This review will focus on these recent advances, emphasizing ZC3H12A’s integrative functions in disease mechanisms and its application prospects in targeted therapies.

## 2. ZC3H12A as the “Master Switch” of Immune Homeostasis

ZC3H12A is a key protein with RNA-binding activity and ribonuclease function, performing extensive regulatory roles in the immune system that extend beyond conventional mRNA degradation [32]. It plays a central role in the cross-network between innate and adaptive immunity by precisely regulating the expression of inflammatory factors and immune-related genes at the post-transcriptional level, maintaining immune homeostasis through multiple mechanisms (Figure 2).

### 2.1. Regulatory Functions in Innate Immunity

Innate immune cells, such as macrophages, neutrophils, and natural killer (NK) cells, recognize pathogen-associated molecular patterns (PAMPs) and damage-associated molecular patterns (DAMPs) through pattern recognition receptors (PRRs) expressed on their surface or intracellularly [33]. In macrophages, ZC3H12A exerts a crucial “braking” function by degrading mRNA of key inflammatory factors such as *IL-6*, *IL-12b*, and *TNF-α*, thereby limiting excessive activation of M1 polarization. Studies indicate that ZC3H12A deficiency significantly enhances mRNA stability of these cytokines, triggering cytokine storms and systemic inflammation [15,34,35,36]. Concurrently, ZC3H12A plays a crucial role in tissue repair and inflammation resolution by suppressing NF-κB and JNK/c-Myc signaling pathway activity, thereby promoting macrophage polarization toward the M2 type [14,15,37]. Notably, ZC3H12A expression itself is tightly regulated. Inflammatory mediators such as IL-1 and IL-17 can induce ZC3H12A expression by releasing its mRNA translation inhibition, forming a negative feedback regulatory loop [37,38,39,40]. In neutrophils, ZC3H12A participates in regulating the apoptosis process, influencing the resolution of inflammatory responses. In inflammatory bowel disease (IBD) models, its expression levels are closely correlated with pathological indicators such as myeloperoxidase (MPO) activity, reactive oxygen species (ROS) production, and cell migration [41,42]. Clinical observations indicate that elevated expression of ZC3H12A in neutrophils of patients with acute infections correlates with the severity of damage to target organs such as the liver [43]. In NK cells, deficiency of ZC3H12A enhances OCT2/IκBζ–NF-κB complex-mediated transcription of IFN-γ, thereby strengthening antitumor immune responses [44,45,46,47]. Recent studies have revealed that ZC3H12A also serves as a key molecule regulating the pro-fibrotic function of type II innate lymphoid cells (ILC2s). By degrading the mRNA of transcription factors *Gata3* and *Egr1*, it indirectly suppresses the production of pro-fibrotic cytokines such as *IL-5* and *IL-13*, thereby limiting the progression of idiopathic pulmonary fibrosis (IPF) [48]. Further research has revealed a novel upstream regulatory mechanism: the transcription factor Mef2d acts as a “brake” on ZC3H12A by binding to the *ZC3H12A* locus and suppressing its transcription. When Mef2d is absent, ZC3H12A expression increases, leading to excessive degradation of *IL-33* receptor (ST2) and *Gata3* mRNA, ultimately weakening the activation and fibrogenic capacity of ILC2s. This regulatory axis—from Mef2d to ZC3H12A and downstream fibrotic programs—provides a novel perspective on the immunopathological mechanisms of fibrotic diseases and suggests this pathway as a potential therapeutic intervention target [49].

### 2.2. “Brakes and Accelerator” of Adaptive Immunity

Within the adaptive immune system, ZC3H12A exhibits complex bidirectional regulatory properties, functioning both as a “brake” to prevent excessive immune activation and as an “accelerator” to promote effective immune responses under specific conditions. In B cells, ZC3H12A deletion enhances proliferation and class-switch recombination, resulting in severe immunopathology, indicating its fundamental role in preventing B cell-dependent autoimmunity [50]. In T cells, ZC3H12A prevents the generation of abnormal effector CD4 + T cells by targeting mRNAs encoding transcription factors (c-Rel), surface molecules (*Ox40*), and cytokines (*IL-2*) [51]. T-cell receptor (TCR) signaling dynamically regulates ZC3H12A through Malt1-mediated cleavage, facilitating full T-cell activation [51]. Furthermore, the regulatory network formed between ZC3H12A and the RNA-binding protein Roquin is of particular interest. These two proteins physically interact with each other, and disrupting this interaction enhances the effector function and tumor accumulation capacity of tumor-specific T cells [52]. Roquin deficiency primarily impacts Tfh cell differentiation and humoral immunity, whereas ZC3H12A deficiency more readily induces Th17-mediated inflammatory responses. Further knockdown of ZC3H12A in a Roquin-deficient background continues to enhance Th17 differentiation, indicating that the two proteins have non-overlapping regulatory functions [53]. This indicates that ZC3H12A may serve as a potential target for regulating T cell activity. Currently, an anti-degradation mutant of ZC3H12A has been developed. However, it suppresses *TCR-β* expression and signaling, thereby impairing positive selection in the thymus and increasing susceptibility of thymocytes to apoptosis. Therefore, when utilizing ZC3H12A to regulate T cell activity, caution is warranted regarding the potential risk of severe lymphopenia [54].

ZC3H12A plays a key role in regulating immune homeostasis by modulating the initiation and resolution of inflammatory responses in innate immune cells, as well as balancing the intensity and specificity of immune responses in adaptive immune cells. Understanding these regulatory mechanisms improves our understanding of the pathogenesis of immune-related diseases.

## 3. Bidirectional Regulation of ZC3H12A in Tumors: From “Tumor Suppressor Gene” to “Therapeutic Lever”

**Figure 3:**
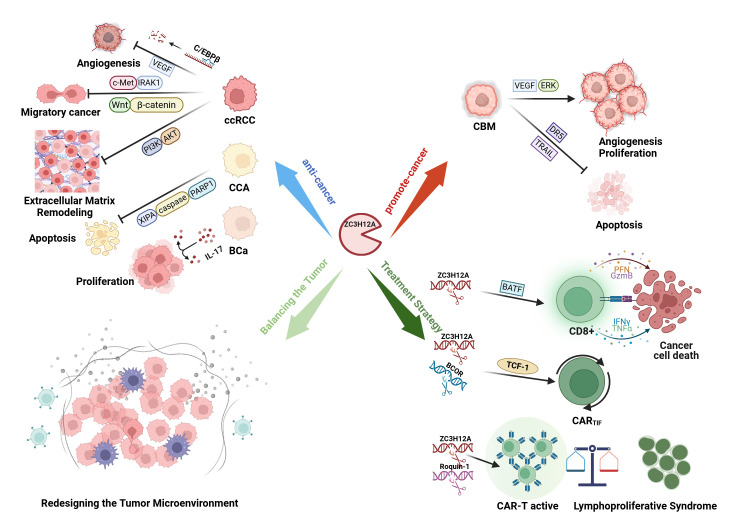
ZC3H12A as a Core Factor in Tumor Regulatory Networks. Under different signaling pathway conditions, ZC3H12A exerts either tumor-suppressive or tumor-promoting effects. In most cancers, such as clear cell renal cell carcinoma (ccRCC), cholangiocarcinoma (CCA), and bladder cancer (BCa), ZC3H12A acts as an oncogene. In colorectal cancer (CRC), it promotes angiogenesis, proliferation, and suppresses apoptosis. *ZC3H12A* prevents tumor immune escape (e.g., in PDAC, GC) by reshaping the immune microenvironment. Combined targeted knockout of ZC3H12A and BCOR enhances the antitumor effects of CART cells and prolongs T cell lifespan, generating CAR_TIF_ cells. Co-targeting with Roquin-1 also boosts antitumor efficacy, though this requires careful management of associated autoimmune toxicity.

ZC3H12A contributes to antitumor immunity and influences multiple biological properties of tumor cells. As a central regulator within the cytokine network, it participates in tumorigenesis and progression through mechanisms including gene expression regulation and miRNA modulation and is closely associated with the homeostasis of the tumor microenvironment (Figure 3) [31,55].

### 3.1. The Intrinsic “Environment-Dependent” Role of Tumor Cells

ZC3H12A exhibits highly context-dependent roles in cancer biology, functioning as either a tumor suppressor or an oncogene depending on the specific cellular signaling environment. Its varying activity across different malignant tumors demonstrates this duality.

In most cancers, ZC3H12A exerts potent tumor suppressing effects by targeting key signaling pathways. In clear cell renal cell carcinoma (ccRCC), angiogenesis is a critical driver of cancer progression [56]. *ZC3H12A* inhibits angiogenesis in ccRCC by degrading transcription factor *C/EBPβ* and angiogenic factors such as *VEGF* and *IL-8* through its RNase activity [57,58,59,60]. Simultaneously, its function is also achieved through negative regulation of the *c-Met*/*IRAK1* signaling axis. When ZC3H12A expression is downregulated—particularly in TKI-resistant states—*IRAK1* activity is enhanced, accelerating ZC3H12A protein degradation and releasing inhibition of the c-Met receptor. This process drives epithelial–mesenchymal transition, angiogenesis, and stemness maintenance [61,62,63]. Its mechanism of action is further reflected in the regulation of the *c-Met*/*CD44* signaling axis: ZC3H12A exerts its effects by negatively regulating the stem cell marker *CD44* (a key co-receptor for c-Met). Deletion of ZC3H12A elevates *CD44* levels, enhances c-Met phosphorylation, and activates downstream pathways, thereby driving tumorigenesis and stemness maintenance. This highlights its function as deeply embedded within the ccRCC-specific signaling network [64]. Additionally, ZC3H12A suppresses the development of clear cell renal cell carcinoma (ccRCC) by inhibiting the *Wnt*/β-catenin signaling pathway. It achieves this by degrading specific miRNAs (miR-519a/b-3p and miR-520c-3p), thereby releasing their suppression of *Wnt* pathway inhibitors (*SFRP4*, *KREMEN1*). This stabilizes the β-catenin degradation complex, ultimately inhibiting β-catenin nuclear translocation and epithelial–mesenchymal transition (EMT) [65]. Its effects also extend to regulating the *PI3K*/*AKT* signaling axis and extracellular matrix remodeling by positively modulating *PTEN* and maintaining *RECK* and *TIMP3* levels, which collectively suppress excessive *PI3K*/*AKT* activation and *MMP* activity [63,66]. In cervical cancer, ZC3H12A binds to *XIAP* mRNA via its ZF domain and promotes its degradation through its PIN domain RNase activity, thereby inducing apoptosis via the *XIAP*/caspase/*PARP1* pathway [67]. Similarly, in breast cancer, ZC3H12A inactivates *IL-17* signaling to suppress tumor growth and metastasis [68] and exerts anti-proliferative effects by inducing G0/G1 cell cycle arrest in triple-negative breast cancer (TNBC) [69].

In gliomas, ZC3H12A promotes angiogenesis through *VEGFA*-mediated *ERK* activation, demonstrating pro-tumor activity. Its knockdown suppresses glioma growth and angiogenesis in vivo [70]. Additionally, ZC3H12A also regulates apoptosis by promoting death receptor 5 (DR5) degradation via the autophagy-lysosome pathway, reducing cellular sensitivity to TRAIL- or DR5-mediated apoptosis [71]. Its expression level negatively correlates with sensitivity to DR5-induced apoptosis, highlight its role in promoting tumor immune evasion and survival [71,72]. Recent studies have explored the implications of ZC3H12A-mediated *NF-κB* inhibition in tumor progression and therapy resistance [73,74].

The dual nature of ZC3H12A highlights its function as a pivotal RNA regulatory hub—its ultimate biological effects are not intrinsic properties but are determined by integrated signaling pathways within specific cellular environments. Its capacity to either suppress or promote tumorigenesis depends on which dominant oncogenic pathways (such as *c-Met*, *Wnt*, *IL-17*, and *VEGFA*/*ERK*) are active and subject to its post-transcriptional regulation (Figure 3).

### 3.2. The “Architect” of the Tumor Immune Microenvironment

Dysfunction of ZC3H12A profoundly reshapes the entire tumor ecosystem. In pancreatic ductal adenocarcinoma (PDAC), IL-1β induces downregulation of ZC3H12A expression, leading to the accumulation of key chemokines and cytokines such as *CXCL1*, *CXCL2*, *CSF2*, and *TGFβ*. This recruits large numbers of myeloid-derived suppressor cells (MDSCs), which inhibit the antitumor function of cytotoxic T lymphocytes (CTLs) and mediate immune escape in PDAC [75]. Similarly, in gastric cancer (GC), dysregulation of the linc00936/miR-425-3p/ZC3H12A axis similarly suppresses the antitumor immunity of CIK cells and CD4^+^ T cells by promoting the secretion of immunosuppressive factors such as *VEGF*, *IL-10*, and *TGF-β1*, ultimately leading to immune escape and disease progression [76]. These two cases highlight the pivotal role of ZC3H12A in sustaining antitumor immune responses.

In contrast, the tumor-suppressing function of ZC3H12A in colorectal cancer (CRC) exhibits a more complex hierarchy. On one hand, epithelial cell-autonomous mechanisms play a dominant role: ZC3H12A in intestinal epithelial cells negatively regulates the *IL-17* signaling pathway by directly degrading *NF-κB IZ* (*IκBζ*) mRNA, thereby suppressing gut microbiota-triggered tumor cell proliferation driven by ERK phosphorylation [77]. On the other hand, the tumor microenvironment also actively participates in attacking ZC3H12A: M2 macrophages deliver miR-143-3p to cancer cells via secreted extracellular vesicles. By targeting and inhibiting ZC3H12A, they release its suppression of the transcription factor *C/EBPβ*, thereby activating pro-cancer signaling and promoting CRC progression [78].

As a pivotal molecular node, the functional state of ZC3H12A directly determines the equilibrium of the tumor ecosystem. Whether driving immune evasion in pancreatic and gastric cancers or mediating cross-talk between epithelial self-defense and microenvironmental signals in colorectal cancer, ZC3H12A inactivation consistently propels more malignant tumor phenotypes. Thus, reshaping the tumor ecosystem via ZC3H12A may emerge as a promising cross-cancer therapeutic strategy.

### 3.3. The “Game Changer” in CAR-T Immunotherapy

In solid tumor therapy, the key to the success of engineered T cells lies in their persistence—that is, their ability to maintain a population of T cells with stem-like properties and self-renewal capacity (such as *TCF-1*^+^ TPEX) within the tumor microenvironment characterized by chronic antigen exposure and metabolic stress [79]. Research indicates that knocking out *ZC3H12A* reprograms CD8^+^ T cells into a unique “long-lived effector” state, significantly enhancing their accumulation, persistent survival, and killing capacity at tumor sites [80]. This process is finely regulated by the transcription factor *BATF*, which acts as a molecular “variable resistor” to coordinate the balance between effector differentiation and mitochondrial fitness. Notably, *BATF* deficiency completely reverses the metabolic advantage and persistence phenotype induced by ZC3H12A knockout, revealing the central role of the “ZC3H12A-BATF-mitochondria-OXPHOS” axis in linking T cell functional states to metabolic reprogramming [80]. In CAR-T systems, mechanism studies further revealed that ZC3H12A directly targets *Tcf7* mRNA and inhibits *TCF-1* protein expression. Its knockout significantly expands the TCF-1^+^ TPEX cell pool, enhances memory-like differentiation and secondary response capacity, thereby markedly improving tumor clearance efficacy and long-term persistence of CAR-T cells in B-ALL models [81].

The most groundbreaking advancement stems from the combined targeting of ZC3H12A and BCOR. Across multiple CAR-T platforms—including *CD19*, *GD2*, and others—simultaneous knockout of ZC3H12A and BCOR induces the generation of functionally distinct “tumor-immunological fitness” (TIF) T cells [82]. Mechanistically, ZC3H12A and BCOR jointly regulate a “core stemness program” comprising approximately 216 factors, exhibiting complementary functions: ZC3H12A knockout primarily initiates the stemness program but is accompanied by increased cell death, whereas BCOR knockout preferentially promotes TPEX proliferation and survival. Their synergy is crucial for achieving “both stemness and functionality” under chronic antigenic conditions [83]. Leveraging the unique properties of CAR*_TIF_* cells, the study also developed GD2-TIF as a platform for single-dose, long-term delivery of biologics. Its demonstrated persistent in vivo persistence and sustained secretion of therapeutic molecules highlight the central role of the ZC3H12A/BCOR axis in coordinating the “stemness-function-metabolism” network and its clinical translational potential [84].

However, safely knocking out ZC3H12A and BCOR has become critical. Traditional CRISPR-Cas systems generate double-strand breaks (DSBs) associated with genomic rearrangements and genotoxicity during multi-site knockouts, inducing programmed cell death [85,86,87]. New research reveals that employing an “orthogonal CRISPR-Cas” strategy enables DSB-free knockout of ZC3H12A and B2M. Simultaneously integrating CAR targeting into the T cell receptor α constant (TRAC) locus significantly reduces the risk of chromosomal translocation (approximately 210-fold), providing a safe and robust manufacturing platform for multi-site engineering [88]. These advances, together with the signals of long-term complete remission observed in preclinical studies, collectively underscore the critical value of “persistence engineering” in overcoming treatment bottlenecks for solid tumors [89].

In solid tumor models treated with humanized engineered T cells, dual knockout of ZC3H12A and Roquin-1 demonstrated stronger antitumor efficacy compared to single knockout. However, some cases developed lymphoproliferative syndrome and related toxicities, suggesting that enhanced therapeutic effects coexist with autoimmune risks [90]. Under these circumstances, ZC3H12A/BCOR dual-targeted CAR*_TIF_* cells can serve as the core foundation. After thoroughly evaluating the risk-benefit ratio, the combination intervention with Roquin-1 should be prudently considered to balance therapeutic gains against the risk of autoimmune toxicity [82,91]. In vivo CRISPR screening studies confirm that knocking out ZC3H12A or Roquin-1 confers significant expansion advantages to CAR-T cells during early treatment phases. These findings also suggest that the impact of different genetic perturbations on persistence exhibits time- and context-dependent effects, necessitating a balance between therapeutic efficacy and toxicity control [92]. To achieve safe and effective translational application, a transgenic expression system can be designed based on the recognition mechanism of endogenous mRNA 3′ UTR stem-loop structures by ZC3H12A/Roquin-1. This system would be suppressed during quiescence and de-inhibited upon T cell activation, thereby specifically enhancing effector function within the tumor microenvironment (Figure 3) [93].

In summary, by leveraging ZC3H12A/BCOR dual-targeted CAR*_TIF_* cells as a core platform, prudently integrating enhanced strategies targeting Roquin-1 under clearly defined risk thresholds, and complementing these with precise transgenic regulatory approaches, we anticipate developing next-generation T-cell therapies for tumor treatment that combine sustained efficacy with safety [84,92,93].

## 4. ZC3H12A-Mediated Systemic Metabolic–Immune Dialog

Nutritional excess and dietary imbalances resulting from modern lifestyles trigger systemic chronic low-grade inflammation through multiple pathways, including lipotoxicity, oxidative stress, and enteric signals. This inflammation subsequently drives metabolic disorders within the pancreas-liver-adipose tissue axis and the onset of related endocrine diseases, forming the key pathological basis of metabolic syndrome [94]. ZC3H12A has been identified as a central node within the intricate network of physiological and pathological processes that make up the “nutrition-inflammation-metabolism” interaction hub.

ZC3H12A expression in subcutaneous adipose tissue exhibits an inverse relationship with BMI. Its suppression in 3T3-L1 adipocytes reduces *GLUT4* expression and diminishes glucose uptake, indicating a fundamental role in sustaining adipocyte metabolic competence and insulin sensitivity [95]. In pancreatic β-cells, ZC3H12A fine-tunes stress-responsive transcriptional networks involving *FOXO1* and *PDX1*; impaired insulin secretion upon its downregulation underscores its importance in β-cell functional integrity [96,97].

Clinical observations link serum ZC3H12A levels to diabetic nephropathy progression. Mechanistically, central dapagliflozin administration elevates hypothalamic ZC3H12A, mitigating neuroinflammation and improving renal lipid metabolic reprogramming—supporting its dual role as a biomarker and therapeutic target within the renal-brain axis [98].

Immunometabolically, myeloid-specific ZC3H12A deletion triggers systemic inflammation and perturbs hepatic metabolism, illustrating its homeostatic function across tissues [99]. Consistent with this, reduced ZC3H12A in obese individuals’ mononuclear cells accompanies elevated inflammatory markers, reinforcing its protective role against meta-inflammation [100]. Notably, the ZC3H12A/BCOR-dependent T cell platform permits persistent metabolic modulator delivery, evidenced by GLP-1-secreting GD2-TIF cells thwarting obesity and diabetes in vivo—validating cell-based therapeutic strategies for sustained metabolic-inflammatory rebalancing [84].

In summary, ZC3H12A constitutes a molecular linchpin connecting nutritional excess, metabolic dysfunction, and chronic inflammation. Through its coordinated regulation of adipocyte insulin response, β-cell secretion, and renal metabolic-inflammatory coupling, it articulates core mechanisms of metabolic syndrome. Targeted modulation of ZC3H12A thus offers a compelling strategy for stratified metabolic intervention, spanning lifestyle, hormonal, immunometabolic, and cell-based modalities.

## 5. New Strategies for Treating Inflammatory and Infectious Diseases with ZC3H12A

### 5.1. Enhancing ZC3H12A as a Therapeutic Strategy in Inflammatory Diseases

**Figure 4:**
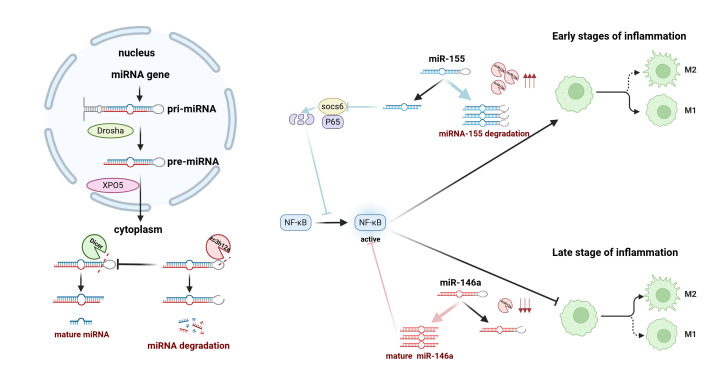
Schematic illustration of how ribonuclease ZC3H12A cleaves miRNA antagonizing Dicer and achieves precise regulation at different stages of inflammation. *ZC3H12A* directly targets pre-miRNAs, cleaving the terminal loop of pre-miRNAs to antagonize Dicer nucleases and inhibit their maturation, thereby reducing the production of mature miRNAs and affecting their activity. In the early stages of inflammation, macrophages polarize toward the M1 phenotype. In response to inflammatory stimuli, miR-155 is highly transcribed and negatively regulates SOCS6, which degrades p65 through ubiquitination, thereby inhibiting the NF-κB signaling pathway and promoting the initiation of the inflammatory response. ZC3H12A upregulates miR-155 expression in the early stages and inhibits maturation. In the late stages of inflammation, miR-146a gradually accumulates and suppresses NF-κB activation by inhibiting IRAK1 and TRAF6 expression. This promotes polarization of macrophages from M1 to M2, contributing to the termination and resolution of the inflammatory response. At this stage, ZC3H12A is downregulated, increasing the maturation of miR-146a and inhibiting NF-κB activity. Therefore, dynamic regulation of ZC3H12A during inflammation is key to the resolution of inflammation.

ZC3H12A is a ribonuclease that directly impairs microRNA (miRNA) maturation through cleavage of precursor miRNAs (pre-miRNAs), thereby directly regulating the microRNA maturation process. By antagonizing the Dicer nuclease, it exerts a broad influence on microRNA-mediated gene silencing [101,102,103,104]. In the early stages of inflammation, ZC3H12A regulates inflammatory responses by suppressing the miR-155/RORα axis and preventing excessive NF-κB signaling activation (Figure 4) [105]. Conversely, its downregulation during the late inflammatory phase promotes miR-146a maturation, thereby facilitating inflammation resolution [106,107]. In addition to regulating miRNAs, ZC3H12A can directly degrade the mRNA of various inflammatory mediators, such as IL-6. This has anti-inflammatory and tissue-protective effects in multiple organs, including the cardiovascular system, lungs, liver, kidneys and intestines [108,109,110,111,112,113,114]. In recent years, therapeutic strategies targeting the activation of ZC3H12A have made significant progress. Research indicates that ZC3H12A recognizes stem-loop structures within the 3′ UTR regions of inflammation-related mRNAs, including its own mRNA, and directs their degradation [115]. Based on this mechanism, researchers designed antisense morpholino oligonucleotides (MOs) targeting this autoregulatory site. By specifically blocking ZC3H12A’s degradation of its own mRNA, these MOs elevated its protein expression levels, thereby enhancing its anti-inflammatory function. Crucially, this approach did not impair its ability to recognize other target mRNAs such as *IL-6*, offering a novel nucleic acid therapeutic strategy for treating inflammatory diseases [116].

### 5.2. The Dual Role of ZC3H12A in Infectious Diseases

Although the absence of ZC3H12A in infectious diseases may exacerbate autoimmune responses, it may impair host defense in some infections; for example, ZC3H12A knockout enhances type I interferon responses and improves bacterial clearance in Klebsiella pneumoniae (KP) infection [117]. Notably, ZC3H12A directly targets viral RNAs; it degrades spliced HIV-1 transcripts to suppress replication [118,119], cleaves non-ARE regions in coxsackievirus B3 RNA [120], degrades KSHV pre-miRNAs, suppresses Dicer [121], modulates miR-122 biogenesis to inhibit HCV [122], and accelerates HBV RNA decay via recognition of the terminal redundancy region [123]. In chronic viral infections, co-inhibition of BCOR and ZC3H12A promotes stem-like exhausted T cells and improves viral control [83].

Therefore, increasing ZC3H12A activity could be useful in treating inflammatory diseases, while temporarily inhibiting it could help defend against certain infections, demonstrating its context-specific therapeutic potential.

## 6. Conclusions and Perspectives

In summary, ZC3H12A, as a key regulatory factor with both RNase and deubiquitinating enzyme activities, plays a central role in immune homeostasis, inflammatory responses, and tumorigenesis. In recent years, its breakthrough progress in CAR-T cell therapy has been particularly noteworthy: knocking out ZC3H12A significantly enhances CAR-T cell persistence, stem cell-like properties, and anti-tumor capabilities. Notably, the strategy of co-knocking out ZC3H12A and BCOR successfully induces CAR_TIF_ cells with “tumor immune adaptability,” demonstrating potential for long-term survival and sustained tumor clearance. Despite safety challenges, novel gene editing strategies and precision regulatory systems offer viable pathways for clinical application. Consequently, ZC3H12A has emerged as a key molecular target for optimizing T-cell therapies and advancing tumor immunotherapy.

Moreover, its potential involvement in DNA damage repair remains less explored. DNA damage triggers NF-κB activation, which contributes to cellular response to genotoxic stress. Previous studies indicate that TANK, ZC3H12A, and USP10 can form a deubiquitination complex that suppresses genotoxic NF-κB activation by reducing the ubiquitination levels of TRAF6 and NEMO [29]. This mechanism may help overcome resistance to cancer therapies and alleviate autoimmune pathologies. Recent work from our laboratory identified ZC3H12A as a factor influencing DNA damage repair and radiosensitivity in small cell lung cancer (SCLC) [124]. Future studies should aim to elucidate the specific molecular mechanisms by which ZC3H12A affects DNA damage repair, as well as the precise regulation of ZC3H12A activity and the development of targeted therapeutic agents.

## Data Availability

No new data were created or analyzed in this study.

## Abbreviations

SL	stem loop
TLRs	Toll-like receptors
PRRs	pattern-recognition receptors
RBPs	RNA-binding proteins
miRNAs	microRNAs
3′ UTR	3′ untranslated region
DUB	deubiquitination enzyme
NF-κB	nuclear factor kappa-B
IHD	ischemic heart disease
MCP-1	monocyte chemoattractant protein 1
CCR2	monocyte chemokine receptor 2
ESTs	expressed sequence tags
MCPIP	MCP-inducible protein
LPS	lipopolysaccharide
SNPs	single nucleotide polymorphisms
PRDs	proline-rich structural domains
NCD	N-terminal conserved structural domain
UBA	ubiquitin-associated functional domain
JNK	c-Jun N-terminal kinase
TTP	tristetraprolin
ZF	zinc finger motif
DR5	death receptor 5
pri-miRNA	Primary miRNA
pre-miRNA	precursor miRNA
RORα	retinoid-related orphan receptor α
PAMPs	pathogen-associated molecular patterns
IBD	inflammatory bowel disease
NK	Natural killer
OCT2	octamer binding protein 2
TCR	T cell receptor
MOs	morpholino oligonucleotides
KP	Klebsiella pneumoniae
RCC	renal cell carcinoma
ccRCC	clear cell renal cell carcinoma
VEGF	vascular endothelial growth factor
TRAIL	tumor necrosis factor-related apoptosis-inducing ligand
CRC	colorectal cancer
TNF-α	tumor necrosis factor α
ACT	Apheresis cell therapy
CAR	chimeric antigen receptor
CVB3	coxsackievirus B3
ARE	AU-rich element
HCV	hepatitis C
TR	terminal redundant
DAMPs	damage-associated molecular patterns
XPO5	Exportin-5
T2DM	type 2 diabetes
DKD	diabetic kidney disease
TKIs	tyrosine kinase inhibitors
C/EBPβ	CCAAT/enhancer-binding protein β
EMT	epithelial–mesenchymal transition
TNBC	triple-negative breast cancer
SCLC	small cell lung cancer
TIF	tumor-immunological fitness
FGF21	fibroblast growth factor 21
ILC2s	type II innate lymphoid cells
PDAC	pancreatic ductal adenocarcinoma
GC	gastric cancer
CTLs	cytotoxic T lymphocytes
